# Monitoring micro-rheological and multi-organ microcirculatory changes in abdominal sepsis in rats – a new approach to analyzing microcirculatory videos

**DOI:** 10.3389/fphys.2026.1816515

**Published:** 2026-05-12

**Authors:** Adam Varga, Tibor Bekesi, Adam Attila Matrai, Laszlo Adam Fazekas, Adam Deak, Balazs Ujhelyi, Istvan Laszlo, Norbert Nemeth

**Affiliations:** 1 Department of Operative Techniques and Surgical Research, Faculty of Medicine, University of Debrecen, Debrecen, Hungary; 2 Department of Anesthesiology and Intensive Care, Faculty of Medicine, University of Debrecen, Debrecen, Hungary

**Keywords:** hemorheology, microcirculation, red blood cell aggregation, red blood cell deformability, sepsis

## Abstract

**Background:**

The hemorheological and microcirculatory aspects of the pathophysiology of sepsis are partially understood and are often controversial in terms of the dynamics, extent, and correlation of parameters. We aimed to simultaneously investigate hemorheological and multi-organ microcirculatory changes in abdominal sepsis in the rat. Sixteen male Wistar rats (body weight: 389.1 ± 44.9 g) were included in our studies. Animals were randomly assigned to sham-operated control (n=8) or sepsis group (n=8). In the sepsis group, sepsis induction was performed by coecum ligation and puncture after median laparotomy. In the sham-operated group, only laparotomy and abdominal wall closure were performed. Body and rectal temperature, respiratory rate, abdominal aortic blood flow (T206 Transonic System) were measured before and after 24 h after surgery, and microcirculatory recordings were captured using a Cytocam-IDF camera in several localizations (skin, coecum, kidney). A scoring system has been developed for the evaluation of the recordings. These video images were evaluated semi-quantitatively based on the deviations (oedema, presence of red blood cell aggregates, flow heterogeneity and vasculature regularity, scored with 0–4 points per category). After blood sampling, we determined hematological, hemorheological, acid-base, and metabolic parameters.

**Results:**

Body and rectal temperatures were significantly elevated in the sepsis group compared to the control group (p=0.005; p=0.016). Major increases in lactate and creatinine concentrations indicated progression of sepsis (p<0.001 vs. baseline). Increased deterioration of microcirculation was observed: the percentage and density of perfused vessels were significantly decreased in sepsis (coecum: p<0.001 vs. baseline). We saw similar results during scoring the videos, with heterogeneous blood flow and elevated number of aggregates. Erythrocyte aggregation parameters were increased in the sepsis and control group (p<0.001 vs. baseline) and erythrocyte deformability showed a significant decrease (EI_max_: p=0.012 vs. baseline).

**Conclusion:**

The micro-rheological parameters of erythrocytes and microcirculatory changes can majorly impact tissue perfusion during sepsis. The analysis of microcirculatory recordings and the new scoring system can provide useful information on the impact of sepsis on tissue microcirculation.

## 1 Introduction

Sepsis is a life-threatening organ dysfunction caused by the body’s uncontrolled response to infection in most cases. According to a 2020 study, in 2017, there were an estimated 48.9 million cases worldwide, with severe sepsis cases accounting for nearly half of this number and mortality estimated at 11 million (Singer et al., 2016; Rudd et al., 2020). Sepsis is not a disease; it is a syndrome. The primary clinical manifestations include fever or hypothermia, tachycardia, and an increased respiratory rate, accompanied by either leukocytosis or leukopenia. These signs reflect a systemic inflammatory response to infection. In more advanced or severe cases, the condition may progress to hypotension due to circulatory dysfunction, which indicates the potential development of sepsis or septic shock. In addition to clinical signs, confirmation by biochemical markers is essential, but it is important to note that there is no gold standard diagnostic test that can confirm the presence of sepsis 100% of the time.

The definition of sepsis has changed several times over the past decades, and research has been ongoing to come up with the latest definition. The Sepsis-3 definition, introduced in 2016, distinguishes between the following stages of severity: infection, sepsis, septic shock, and multiple organ failure (MOF) [1]. Sepsis, as mentioned above, is a life-threatening organ dysfunction due to infection. Septic shock is a part of sepsis, with circulatory and cellular/metabolic abnormalities and high mortality. The SOFA (Sequential Organ Failure Assessment) scoring system is used to define organ failure (Vincent et al., 1998).

The pathophysiology of the septic process is complex. During infection, pathogen-associated molecular patterns (PAMPs) are recognized primarily by toll-like receptors (TLRs), triggering the innate immune response. In contrast, in non-infectious forms of inflammation and during mechanical trauma, endogenous damage-associated molecular patterns (DAMPs) activate NOD-like receptors, leading to a similar inflammatory cascade. In macrophages, the NF-κB pathway is activated, triggering various cytokine-secreting processes in response, and the cytokines produced can be both pro- and anti-inflammatory. This so-called “cytokine storm” is responsible for the severe consequences. Inflammation causes an imbalance between oxygen and nutrient demand and supply (Jarczak et al., 2021). These are counteracted by compensatory mechanisms. Initially, the concentration of vasodilatory mediators, mainly nitric oxide, increases, leading to a transient enhancement of tissue perfusion for a certain time. This phase is followed by mitochondrial dysfunction, marking the onset of the mitochondrial phase of the pathophysiological process (Hu et al., 2024). It can be assumed that the enzymes of terminal oxidation are inhibited in mitochondria to save energy, but the amount of reactive oxygen and nitrogen derivatives increases as a consequence. Blood vessels are damaged and capillary barrier functions are impaired by oxidative stress. The capillary dysfunction that occurs centers on deteriorating microcirculatory processes, and sepsis damages red blood cells through inflammation and oxidative stress, making them more rigid and less able to pass through small blood vessels. This reduces oxygen delivery to tissues and contributes to organ failure. It also triggers eryptosis, a programmed form of RBC death, decreasing the number of healthy cells, which can lead to increased red blood cell aggregation, increased plasma and whole blood viscosity, and decreased red blood cell deformability (Sordia et al., 2006; Nemeth and Fulesdi, 2016). Additionally, impaired RBC function affects oxygen release, while their breakdown and reduced production often lead to anemia. Although RBCs can interact with pathogens, they may also worsen inflammation during sepsis (Nicolescu and Moldovan, 2024).

Red blood cell deformability is essential for the normal functioning of the microcirculation. This shear-induced passive deformability is necessary to allow red blood cells to pass through smaller diameter blood vessels. Red blood cells are characterized by their biconcave cell shape, resembling a doughnut. At lower velocity gradients they retain their shape, whereas at higher velocity gradients they take on an ellipsoidal shape (Baskurt, 2007). Due to the reduced deformability in sepsis, the red blood cells lose their ability to change shape and may even block capillaries. Cytokines and endotoxins damage endothelial cells, resulting in vasodilation, increased permeability, and edema formation. Flow heterogeneity develops: the local hypoperfusion is accompanied by compensatory hyperperfusion, leading to tissue hypoxia. The deterioration of microcirculation may also be contributed to by activation of the coagulation system, leading to the formation of microthrombi. It is assumed that the production of NO causes the capillaries to dilate, resulting in increased fluid in the blood vessels. However, the barrier functions of the capillaries are impaired, and fluid leaks out of the blood vessels into the interstitium, resulting in the formation of oedema. These processes lead to inadequate organ function (Voerman et al., 1989; Piagnerelli et al., 2003b; Sallisalmi et al., 2014).

The pathophysiological aspects of the blood flow in sepsis are not yet fully understood and there are several contradictions in the literature regarding the extent and dynamics of the changes and the relationships between the parameters. Based on our previous research, we set as the aim of our experiment to investigate hemorheological and multi-organ microcirculatory changes in abdominal sepsis in the rat, using a modern video microscopy technique and extensive micro-rheological studies. In this study, we aim to examine the mechanisms leading to capillary dysfunction, focusing on changes in red blood cell aggregation and deformability. Using the Cytocam-IDF device, we plan to record microcirculatory images from the skin, coecum, and renal cortex in both control and septic groups to observe visible alterations during the progression of sepsis.

## 2 Materials and methods

### 2.1 Experimental animals, sampling protocol

With proper authorization (Registration No.: 18/2023/UDCAW), sixteen male Wistar rats were randomly assigned to either a sham-operated control group (n=8) or a sepsis group (n=8). Sepsis was induced in the sepsis group using the standard cecal ligation and puncture (CLP) method under general anesthesia with 100 mg/bwkg of ketamine i.p. (CP-ketamine hydrochloride 10%, Produlab Pharma BV, Raamsdonksveer, The Netherlands), 10 mg/bwkg of xylazine i.p. (CP-xylazine hydrochloride, 2%; Produlab Pharma BV, Raamsdonksveer, The Netherlands) (Maier et al., 2004; Poli-de-Figueiredo et al., 2008; Toscano et al., 2011). The surgical area was shaved, sterilized, and isolated, followed by a median laparotomy. The coecum, typically located in the lower right abdominal segment, was identified and isolated. Ligature was placed distal to the ileocecal (Bauhin) valve, ensuring no arterial damage to avoid complications. The ligation happened 0.8-1.2 mm from the Bauhin valve for 24 hours. Single-site perforation was made using a 20 G needle, followed by gentle pressure to release intestinal contents into the peritoneal cavity (Cai et al., 2023). Finally, the abdominal wall was closed in two layers. Fluid replacement was performed using a 0,9% NaCl infusion solution administered via the lateral caudal vein at a dose of 3 ml/kg body weight following the procedure. Skin and rectal temperature, respiratory rate, and abdominal aortic blood flow (T206 Transonic System) was measured before and 24 h after surgery. After blood sampling, we determined hematological, hemorheological, acid-base, and metabolic parameters. The required blood sample was taken from the lateral tail vein using a 26G cannula (0.5 ml of blood coagulated with K3-EDTA). A volume of 0.9% NaCl equal to the amount of blood taken was administered through the cannula. The animals were warmed with a heating pad until they woke up. Pain was alleviated by administration of tramadol (15 mg/kg) after surgical intervention, subcutaneously (Sacerdote, 2006).

### 2.2 Hematological and hemodynamic variables

The quantitative hematological variables were analyzed by Sysmex K-4500 microcell counter (TOA Medical Electronics Co., Ltd., Kobe, Japan). In this study, red blood cell count (RBC [T/L]), white blood cell count (WBC [G/L]), hemoglobin concentration (Hgb [g/dl]), hematocrit (Hct [%]), mean corpuscular volume (MCV [fl]), mean corpuscular hemoglobin (MCH [pg]), mean corpuscular hemoglobin concentration (MCHC [g/dl]), and platelet count (Plt [G/L]) were assessed. The right common carotid artery was prepared and cannulated (O.D. 0.965 mm, Polyethylene Tubing Clay Adams, 427411, BD IntramedicTM, Sollentuna, Sweden). After fixation with a central ligature, the cannula was connected to an invasive hemodynamic monitoring system (Hemosys monitor system LD-01, Experimetria Ltd., Budapest, Hungary).

### 2.3 Red blood cell deformability measurements

RBC deformability was tested using a LoRRca MaxSis Osmoscan ektacytometer (Mechatronics BV, The Netherlands) (Baskurt, 2007; Baskurt et al., 2009a). For the deformability test 10 µl whole blood were gently dispersed in 2 ml of polyvinylpyrrolidone (PVP)–PBS solution (PVP: 360 kDa, Sigma-Aldrich Co. USA; PVP-PBS solution viscosity = 30.4 mPas, osmolality = 302 mOsmol/kg, pH = 7.2). In the device elongation in-dex (EI) was determined in the function of shear stress (SS [Pa], range: 0.3–30 Pa). For the comparison of the EI-SS curves EI values at 3 Pa, maximal elongation index (EI_max_), and the shear stress belonging to the half EI_max_ (SS_1/2_, [Pa]) were used, based on Lineweaver-Burk equation (Baskurt et al., 2009c).

### 2.4 Determination of red blood cell aggregation

In this study, the Myrenne MA-1 erythrocyte aggregometer (Myrenne GmbH, Roetgen, Germany) was used, which works on the principle of light transmission. 20 µl anticoagulated blood sample is dropped onto the center of a glass slide and then the slide is folded over it, so that the sample is spread out on the glass. The lens performs a slow, pre-set movement, causing the red blood cells to disaggregate and the light transmission to decrease. This is called the M1 mode. In the M0 mode, the rotation stops, which causes aggregation of the RBCs, light transmission increases. In both modes, the machine takes measurements at the 5th and 10th second, and from these data an aggregation index value is obtained (Baskurt, 2007; Baskurt et al., 2009a, d, 2014; Vayá et al., 2014).

### 2.5 Venous acid-base and blood gas parameters

The epoc^®^ Blood Analysis System was used to determine blood gas, electrolyte and metabolite values. The epoc^®^ is a point-of-care-testing (POCT) device that allows rapid analysis of blood samples, can measure multiple parameters simultaneously and is easily portable. The device consists of a wireless card reader, a card containing a sensor and a personal data assistant for data analysis. A 0.1 ml blood sample was collected from the lateral tail vein using a 26G cannula. The device can measure a wide range of parameters including Na^+^, K^+^, Ca^2+^, Cl^-^ ions, glucose, creatinine, hemoglobin and lactate concentrations, pH, partial carbon dioxide (*p*CO_2_) and oxygen (*p*O_2_) pressures. Of these, partial carbon dioxide and oxygen pressures, lactate and creatinine concentrations are highlighted.

### 2.6 Microcirculation

Video microscopic images of the skin on the abdomen above the navel, coecum and renal cortex were taken at the time points specified in the experimental protocol to study microcirculation, using the CytoCam-IDF device (Cytocam, Braedius Medical, Amsterdam, NL). The device emitted green light at a wavelength of 540 nm on the tissues. Part of the light is absorbed by the tissue and most of it is reflected, which is detected by the 14 megapixel camera of the CytoCam-IDF and converted into a digital image. The key is the hemoglobin content of the red blood cell, which absorbs the light at the wavelength used. The advantages of the device are compact size, advanced lens optics, high resolution, automatic data analysis (Aykut et al., 2015; Hutchings et al., 2016). We aimed to standardize the measurements across the entire range. To avoid pressure artifacts, we used a custom-designed camera stand to perform the measurements. To maintain uniform tissue perfusion, the measurement areas were treated with a body-temperature physiological saline solution. We took 3 images of each measurement area, each 3 seconds long, with a frame rate of 95 frames per second. During the measurements, the sensor head was covered with a sterile cap, which was replaced between measurements.

Among the parameters that can be measured with the instrument are:

- Perfused vessel density (PVD): vessel density multiplied by the fraction of perfused vessels (mm/mm^2^)
- Proportion of perfused vessels (PPV): the number of perfused vessels divided by the total number of vessels x 100 (%)
- microvascular flow index (MFI): used to visually characterize the flow of blood vessels, the average of the scores is used (a number without units)
- UD Microcirculatory Video Score System (MVSS): A scoring system defined by the Department of Operative Techniques and Surgical Research, University of Debrecen. Video images were evaluated semi-quantitatively based on the deviations (oedema, presence of red blood cell aggregates, flow heterogeneity and vasculature regularity, scored with 0–3 points per category) (**Table 1**).

**Table 1:** UD Microcirculatory Video Score System (MVSS).

Parameter	Score	Description
1. Presence of edema	0	none
	1	mild
	2	extensive edema
2. Red blood cell aggregates in large (non-capillary vessels)	0	none
	1	scattered
	2	moderate
	3	large amount, extensive aggregates
3. Visible red blood cell aggregates in capillaries	0	none
	1	scattered
	2	moderate
	3	large amount, extensive aggregates
4. Flow heterogeneity by observer	0	evenly distributed, dynamic flow
	1	mildly heterogeneous flow, locally slowed dynamics
	2	marked heterogeneity, locally slowed or stagnant flow, alternating flow direction
	3	extensive non-perfused areas, marked stasis, severely impaired microcirculation
5. Vasculature appearance	0	normal structure
	1	locally abnormal geometry
	2	extensive, large focal vasculature markedly abnormal vessel structure

### 2.7 Statistical analysis

The required sample size for the experiment was estimated using Mead’s resource equation method. Data analysis of the experiments was performed using SigmaStat Software 3.1.1.0 (Systat Software Inc., San Jose, CA, USA). After testing the normality of the data distribution by the Kolmogorov–Smirnov test independent/two-sample T-test or the Mann–Whitney rank sum test were used to compare data between control and sepsis groups, and also two-way ANOVA or Kruskal–Wallis’s test was used based on the results of the normality test. The Bonferroni *post hoc* test is used after a primary analysis to identify which specific groups differ significantly. The significance level was determined at p<0.05. Results are presented as mean ± standard deviation (SD). Standardized differences from control values were calculated as the mean MVSS difference between control and sepsis videos, divided by the pooled standard deviation of both groups:


(mean control−mean sepsis)/pooled SD.

The pooled SD is the square root of the mean of the squared SDs of the two groups. This standardized difference indicates how strongly each measurement condition can detect group differences (Baskurt et al., 2009d, b). Tukey’s *post hoc* test is performed after the omnibus test to indicate significant differences among more groups of means.

## 3 Results

### 3.1 Hematological variables

Overall, it can be said that the qualitative and quantitative hematological parameters showed significant changes during our measurements. All values remained in the biologically normal range throughout the experiment. The white blood cell count and red blood cell counts increased significantly in the sepsis group after 24 hours compared to baseline values (WBC: p<0.001 vs. base and vs. control values; RBC: p<0.001 vs. base values) Hematocrit and showed a significant difference in the sepsis group compared to the base values (p<0.001), while MCV parameter increased compared to the control group. In general, it can be assumed that the sepsis group shows a slightly worsening hematological profile (**Table 2**).

**Table 2:** Hematological variables in control group (n=8) and Sepsis group (n=8).

Sampling time	Group	WBC (T/L)	RBC (G/L)	Hct (%)	MCV (fL)	Plt (G/L)
Base	Control	5.35 ± 2.84	8.72 ± 0.41	47.78 ± 1.29	54.78 ± 1.07	780.19 ± 187.56
	Sepsis	4.82 ± 1.89	7.85 ± 0.48	45.04 ± 1.75	55.43 ± 0.77	766.88 ± 92.56
24 h	Control	5.81 ± 2.04	8.81 ± 0.41	48.13 ± 2.10	54.65 ± 0.95	745.43 ± 93.70
	Sepsis	10.23 ± 1.61*#	8.79 ± 0.88*	50.52 ± 2.32*	57.39 ± 1.15#	796.36 ± 128.67

WBC, white blood cell count; RBC, red blood cell count; Hct, hematocrit; MCV, mean corpuscular volume; Plt, platelet count. Data are presented as mean ± S.D.; p<0.05; * vs. Base; # vs. Control.

### 3.2 Red blood cell deformability

**Figure 1:**
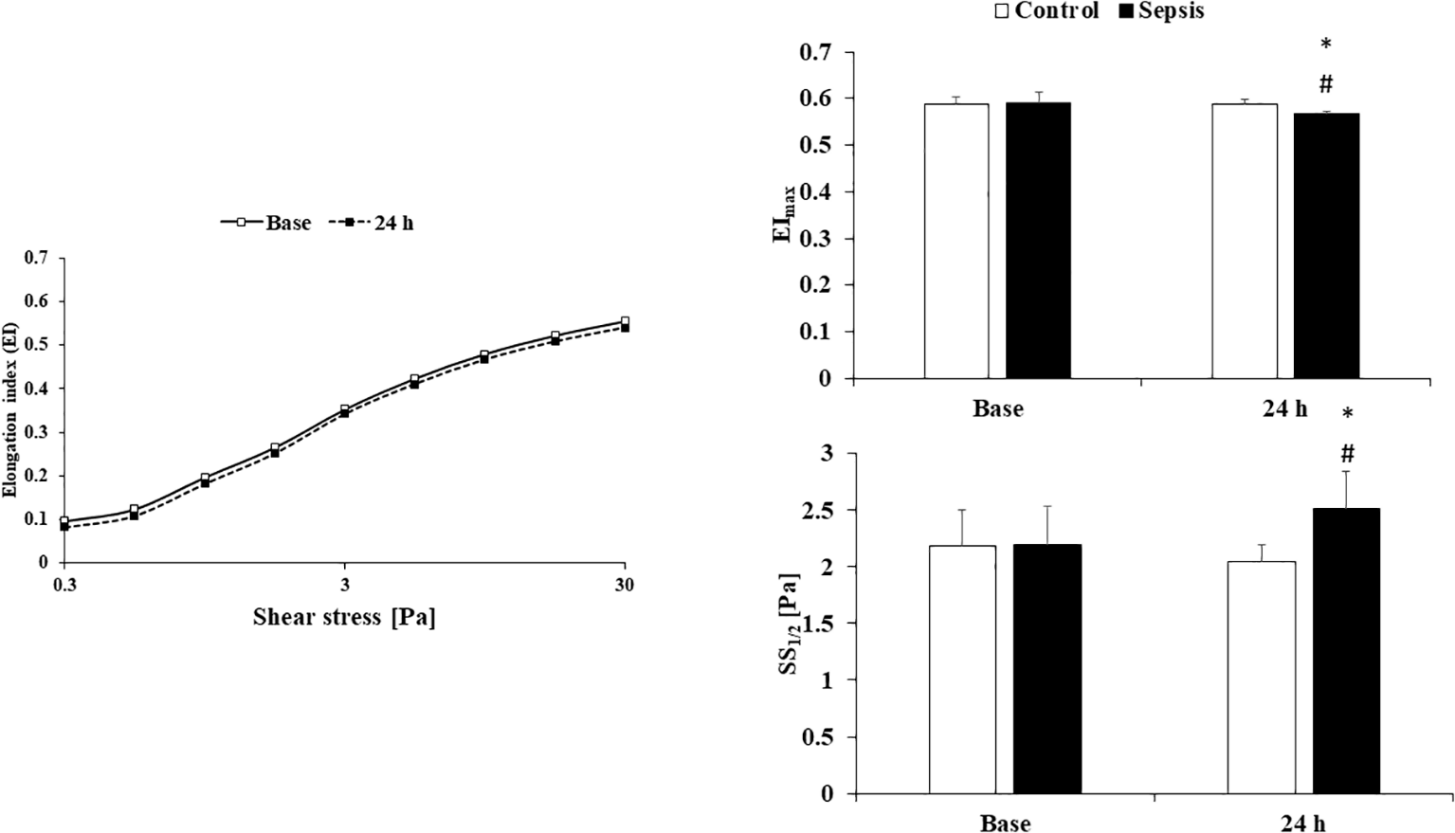
Elongation index in the function of shear stress in the control group (n=8) and sepsis group (n=8) and EI_max_: calculated maximal elongation index - SS_1/2_: shear stress at half EI_max_. Means ± S.D.; *p<0.05 vs. Base; #p<0.05 vs. control.

For the EI-SS curves, the elongation index values at 3 Pa, maximal EI and the SS belonging to the half maximal EI were compared, based on Lineweaver-Burk equation: 1/EI = SS_1/2_/EI_max_ x 1/SS + 1/EI_max_. The elongation index parameter characterizing the deformability of red blood cells can be plotted as a function of the applied shear stress. As shear stress increases, the elongation index of red blood cells increases linearly, as illustrated in **Figure 1**. From the plot of the resulting curve, it can be seen that for the 24-hour measurements, decreased elongation indices were obtained at each measurement point in the sepsis group. By parameterizing the curve, we can obtain additional parameters, such as the SS_½_ value (half of the shear stress associated with EI_max_), whose increase also indicates a deterioration in deformability. As the shear stress increases, the shape of the red blood cells changes from the usual biconcave to an ellipsoidal shape.

### 3.3 Red blood cell aggregation

**Figure 2:**
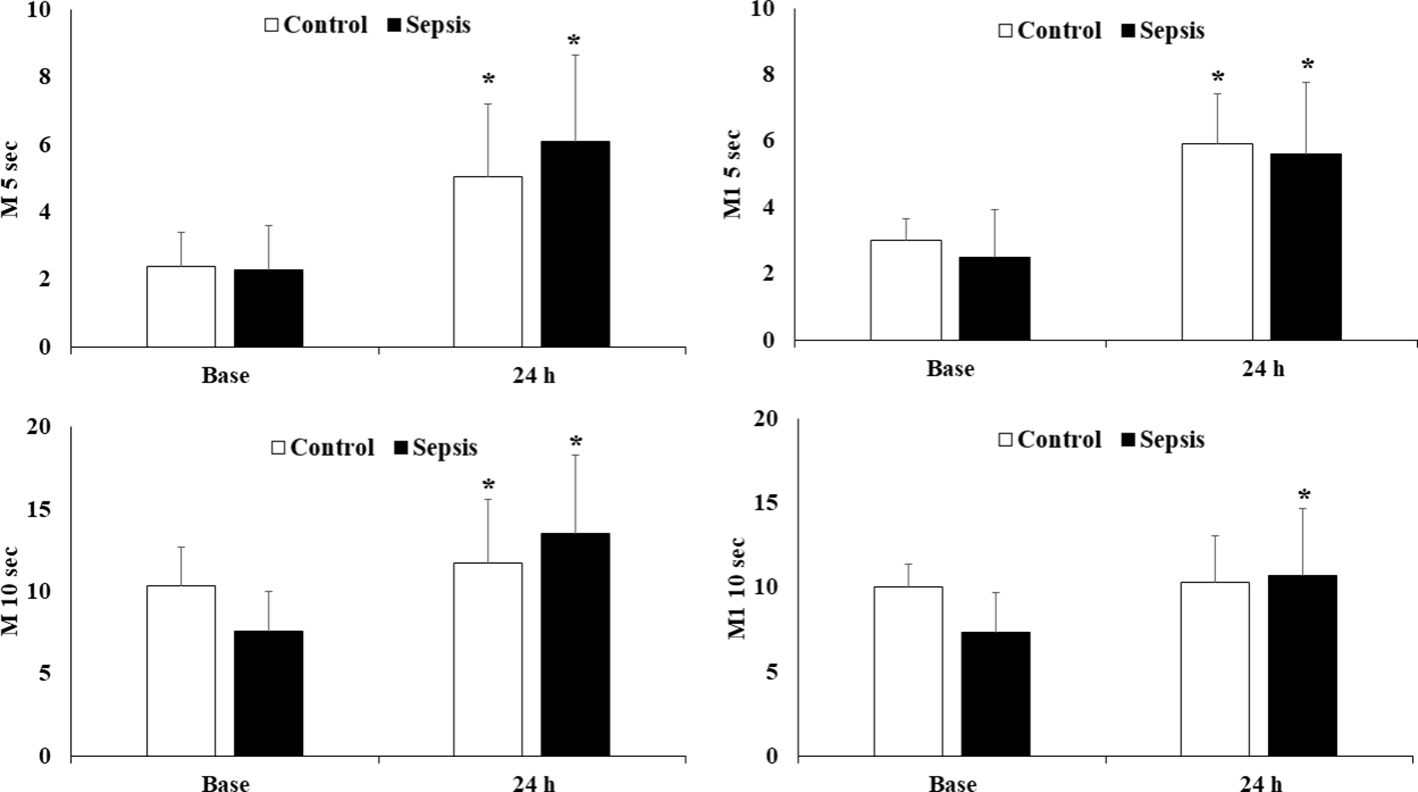
Aggregation parameters of red blood cells (M5 sec, M1–5 sec, M10 sec, M1–10 sec) in control and sepsis group. Means ± S.D.; *p<0.05 vs. Base.

The following **Figure 2**. illustrates the aggregation parameters of red blood cells. The red blood cell aggregation parameters were measured by the light transmission method. When analyzing the 24-hour measurements, we observed that aggregation parameters increased significantly in both the control and sepsis groups. Although there was no significant difference between the two groups after 24 hours, aggregation was slightly more pronounced in the sepsis group for the M 5 sec, M 10 sec, and M1–10 sec parameters.

### 3.4 Venous acid-base and blood gas parameters

**Figure 3:**
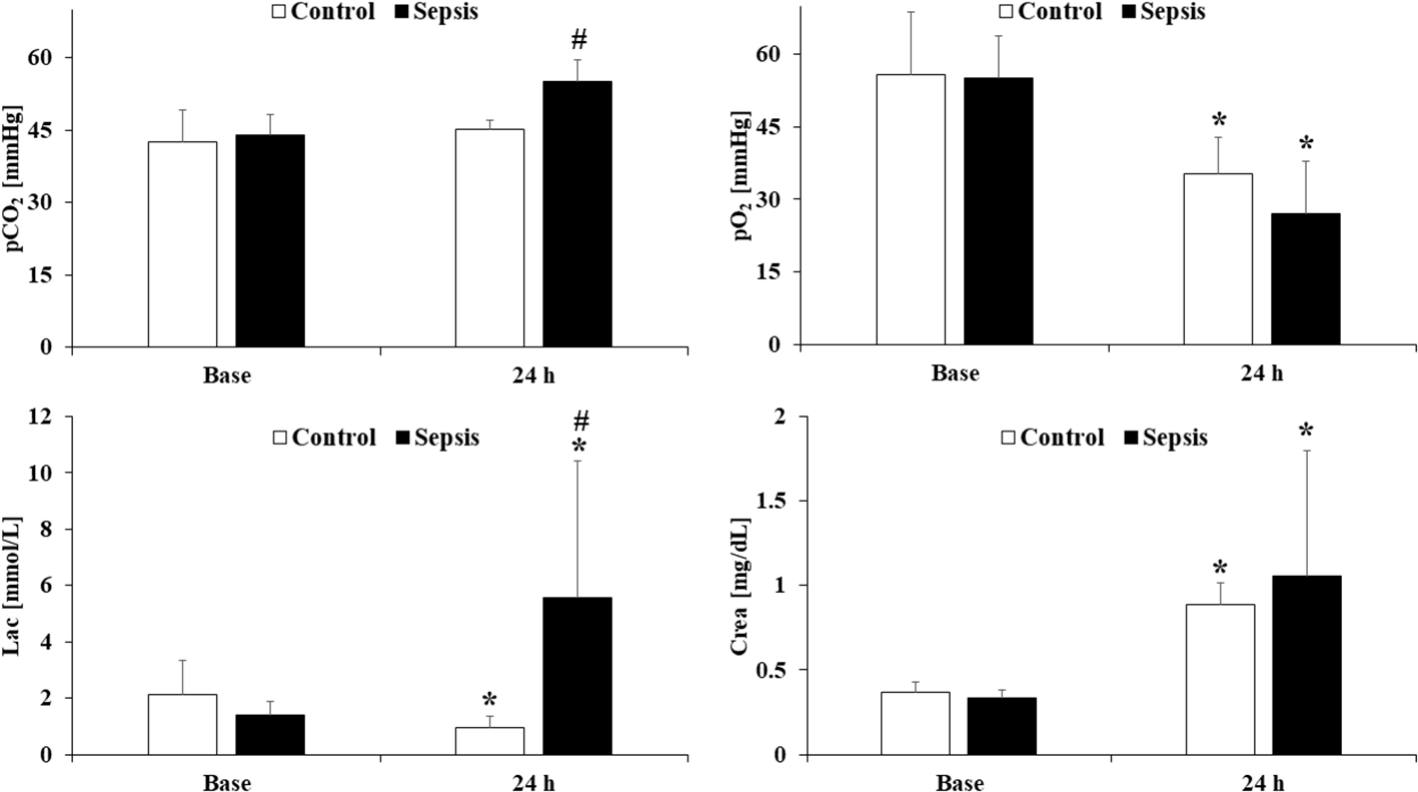
Partial pressure of carbon dioxide, partial oxygen pressure, lactate and creatinine concentration in the control and sepsis group. Means ± S.D.; *p<0.05 vs. Base; #p<0.05 vs. Control.

Among the acid-base and blood gas parameters (**Figure 3**), we would highlight the partial carbon dioxide and oxygen pressures, where significant changes were observed in both groups. Lactate and creatinine concentrations were significantly increased in the sepsis group, being abnormally high after 24 hours.

### 3.5 Hemodynamics and skin temperature

**Table 3** summarizes the hemodynamic parameters and the temperature parameters. Skin and rectal temperatures are shown in the control and sepsis groups before and 24 hours after the intervention. In the 24 h measurements, the body temperature showed an increase in the sepsis group. The aortic flow velocity and heart rate also increased significantly in the sepsis group in the 24 h measurements compared to the control group. In rat models, the modified shock index helps researchers objectively measure the degree of hemodynamic instability in sepsis (Hua et al., 2018). MSI is determined by the simple formula: heart rate/mean arterial blood pressure. Based on the measurements, we observed an elevated shock index in rats in the sepsis group.

**Table 3:** Hemodynamics and body temperatures in control group (n=8) and sepsis group (n=8) in baseline measurements and after one day.

Group		ST (°C)	RT (°C)	BR (b/min)	AF (ml/min)	HR (b/min)	MAP (mmHg)	MSI
Base	Control	35.52 ± 0.18	36.42 ± 0.22	70.5 ± 5.5	4.62 ± 0.77	359.00 ± 22.92	95.5 ± 5.5	3.76 ± 0.23
	Sepsis	35.66 ± 0.46	36.61 ± 0.26	68.5 ± 6.5	4.25 ± 0.65	357.50 ± 24.30	94.6 ± 6.5	3.77 ± 0.22
24 h	Control	35.53 ± 0.23	36.48 ± 0.24	69.8 ± 5.8	4.85 ± 0.54	372.33 ± 31.72	93.4 ± 3.5	3.98 ± 0.18
	Sepsis	37.42 ± 0.42*#	38.68 ± 0.15*#	70.5 ± 4.5	6.81 ± 0.74*#	491.13 ± 13.17*#	65.3 ± 4.2*#	7.55 ± 0.44*#

ST, skin temperature (°C); RT, rectal temperature (°C); BR, breathing rate (breaths per minute); AF, aortic blood flow volume (ml/minutes); HR, heart rate (beats per minute); MAP, mean arterial pressure (mmHg); MSI, modified shock index. Data are presented as mean ± S.D.; p<0.05; * vs. Base; # vs. control.

### 3.6 Microcirculation

**Figure 4:**
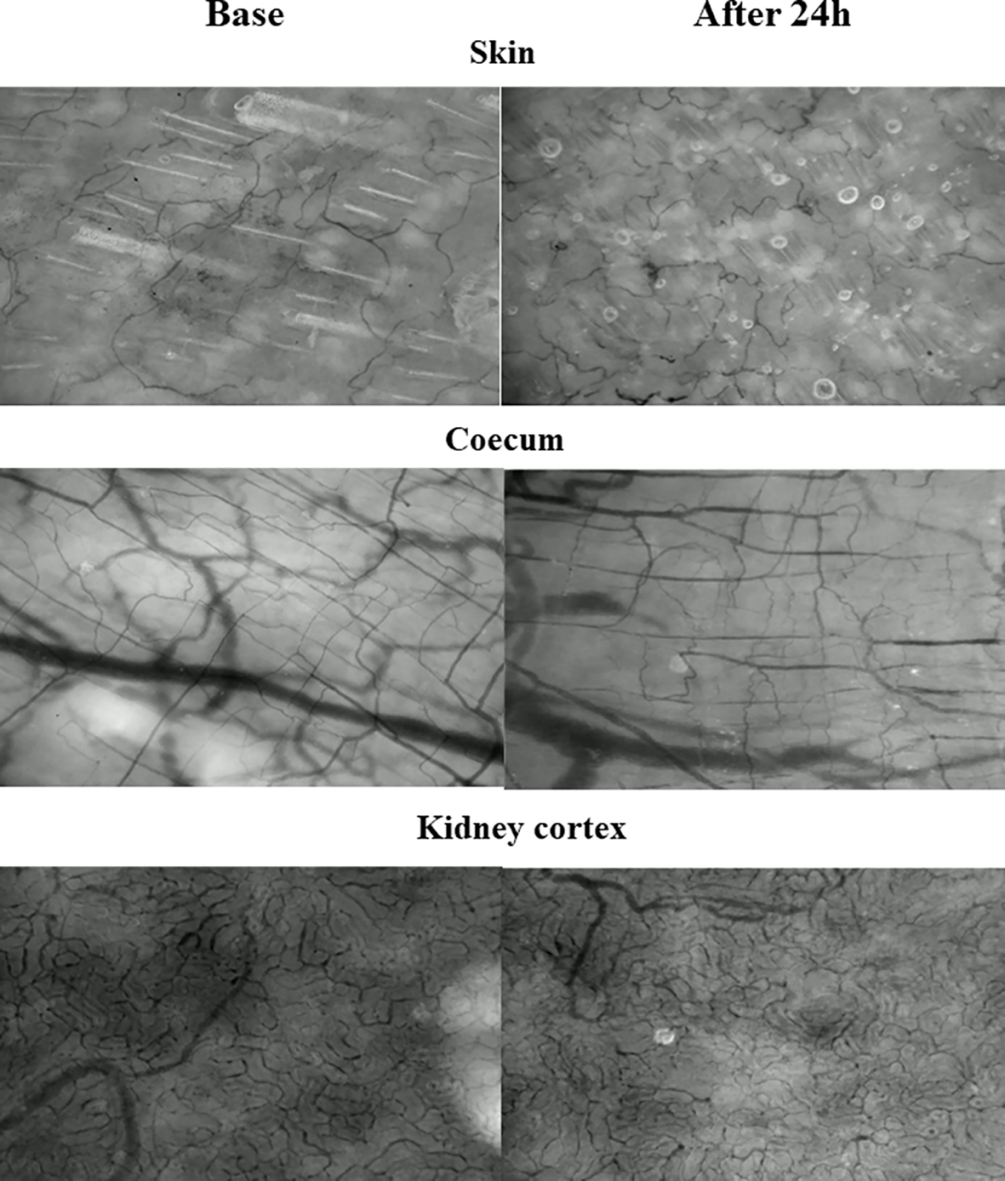
Representative microcirculatory recordings of the different tissues with highlighted abnormalities: **(a)** RBC aggregates in the sepsis group; **(b)** edema in the sepsis group.

Analyzing the microcirculation recordings (**Figure 4**) of the skin in the videos, we observed that the flow in the vessels had slowed down in many cases, stasis was detectable in the capillaries, and the flow pattern was not homogeneous.

A slight decrease was observed in the PVD and PPV values, while the MFI was lower and the MVSS was higher in the septic group (**Table 4**). In contrast, when analyzing the microcirculation recordings of the coecum in the videos, we noticed that the flow in the vessels had often ceased, significant stasis and aggregation were present in many capillaries, and the flow pattern was completely heterogeneous. The PVD, PPV, and MFI values significantly decreased in the sepsis group after 24 hours compared to the control group. The MVSS showed a significant increase in the septic group. Similar microcirculation observations were seen in the renal cortex, where notable edema formation and changes in tissue vascularization were prominent. The density of perfused vessels and the MFI value also decreased in the sepsis group compared to the control group, while the MVSS value significantly increased.

**Table 4:** Microcirculatory parameters of perfused vessel density (PVD), perfused vessel ratio (PPV), microvascular flow index (MFI) and Microcirculatory Video Score System (MVSS).

Site	Group		PVD (mm/mm^2^)	PPV (%)	MFI	MVSS	*Post hoc* test result (MVSS)
Skin	Base	Control	4.25 ± 1.23	5.05 ± 2.55	2.75 ± 0.11	1.04 ± 0.18	–
		Sepsis	4.35 ± 1.03	5.13 ± 2.68	2.78 ± 0.08	0.88 ± 0.26	69.8%
	24 h	Control	4.01 ± 0.89	4.89 ± 3.12	2.72 ± 0.11	0.98 ± 0.25	–
		Sepsis	3.88 ± 0.69	4.65 ± 2.24	2.51 ± 0.06*#	3.43 ± 0.35*#	100%
Coecum	Base	Control	6.35 ± 1.11	55.15 ± 10.26	2.85 ± 0.12	1.25 ± 0.23	–
		Sepsis	6.38 ± 1.28	53.28 ± 12.16	2.79 ± 0.08	1.22 ± 0.25	6.3%
	24 h	Control	6.21 ± 0.76	53.84 ± 12.71	2.78 ± 0.10	1.68 ± 0.29	–
		Sepsis	4.88 ± 0.71*#	41.15 ± 10.15*#	2.32 ± 0.06*#	7.74 ± 0.48*#	100%
Kidney	Base	Control	7.15 ± 1.31	35.25 ± 18.27	2.84 ± 0.13	2.75 ± 0.44	–
		Sepsis	7.28 ± 1.04	33.66 ± 22.27	2.80 ± 0.07	2.48 ± 0.47	53.8%
	24 h	Control	6.81 ± 0.63	33.55 ± 18.41	2.77 ± 0.15	2.68 ± 0.51	–
		Sepsis	6.18 ± 0.52*#	25.14 ± 11.26	2.42 ± 0.09*#	5.19 ± 0.41*#	100%

PVD, perfused vessel density (mm/mm^2^); PPV:proportion of perfused vessels (%); MFI, microvascular flow index; MVSS, Microcirculatory Video Score System; Data are presented as mean ± S.D.; p<0.05; * vs. Base; # vs. control.

**Figure 5:**
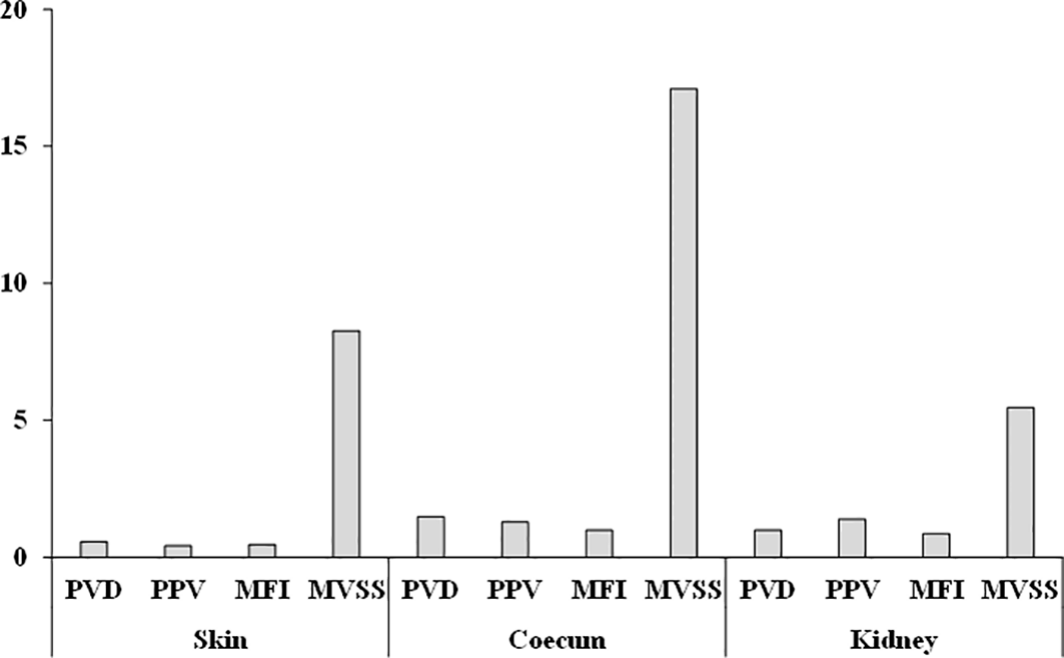
Standardized differences calculated using the data presented in **Table 4**. in the sepsis group after 24 hours.

When determining the standard difference values used in comparing measurement parameters, we observed exceptionally high values for the MVSS parameter we defined compared to the values obtained during automatic analysis with the Cytocam device (**Figure 5**). The most sensitive differences can be seen in the examination of microcirculation in the coecum, followed by measurements of the skin and then the kidney cortex.

## 4 Discussion

Sepsis is a complex, difficult-to-define clinical syndrome characterized by highly variable manifestations, making its definition and early recognition challenging. The severe systemic responses seen in sepsis result from dysregulated inflammatory processes that induce microcirculatory and mitochondrial distress syndrome, as well as capillary dysfunction. In our research, we examined the changes responsible for the development of capillary dysfunction (Yajnik and Maarouf, 2022). Key micro-rheological parameters of red blood cells, such as aggregation and deformability, as well as microcirculatory alterations, can significantly influence tissue perfusion during septic processes (Reinhart et al., 2017; Kotan et al., 2022). The data obtained may provide valuable information for better understanding the relationship between microcirculatory alterations and micro-rheological changes that occur during sepsis (De Backer et al., 2007, 2014; Gruartmoner et al., 2016; De Backer, 2023).

Among the quantitative and qualitative hematological parameters, we examined white blood cell and red blood cell counts, hemoglobin concentration, and the MCV parameter. The normal white blood cell counts in rats range from 6–17 (x10^9^/L). In the sepsis group, we observed elevated values, which can be attributed to inflammation induced by sepsis (Jacob Filho et al., 2018). The normal red blood cell count in rats is 7–10 (x10¹²/L), and our results showed a significant increase in red blood cell count in the septic group. This may be attributed to an initial decrease in red blood cell count driven by inflammation, as well as hypovolemia and dehydration, followed by a compensatory release of mature red blood cells and reticulocytes from the bone marrow into the circulation after 24 hours (Purtle et al., 2017). The normal hemoglobin concentration in male Wistar rats ranges between 10.4–16.5 g/dL. In our results, we observed a marked increase. The normal range of the MCV parameter in Wistar rats is 45–61 fL, and we also observed an increase in this value. In comparison, clinical studies conducted on septic patients reported a decrease in red blood cell count, hemoglobin concentration, and hematocrit values within the first 24 hours, although no significant changes were found in the MCV parameter (Piagnerelli et al., 2003a; Bateman et al., 2017; Delwatta et al., 2018).

In our measurements of red blood cell (RBC) deformability, we found that in the septic group, the elongation index (EI) decreased at every single measurement point after 24 hours. This is clinically significant for us. Experimental studies in rat models have demonstrated that the reduction in red blood cell deformability can be detected within 3–6 hours, while in septic patients, similar process takes place during the first 24 hours of clinical observation (Langenfeld et al., 1991). This information may also help differentiate between trauma and septic patients, as deformability can return to normal levels in trauma patients, while it does not in septic cases. In addition, the degree of reduction in red blood cell deformability shows a correlation with the development of organ failure and the severity of disease outcome. As for red blood cell aggregation parameters, we observed markedly elevated values in the septic group after 24 hours. Previous studies have already examined red blood cell-related changes in sepsis and inflammatory processes. Ko Eunji and colleagues examined the clinical significance of early changes in red blood cell parameters during the initial phase of septic processes. In their study, they used an endotoxin model, inducing sepsis intraperitoneally in rats using the LPS method (Ko et al., 2018). They documented reduced RBC deformability, lower T ½ (sec) values, and increased RBC aggregation parameters. These early alterations in RBC parameters correlated with mortality although the relationship between early red blood cell changes and the clinical symptoms observed in the SOFA scoring system is still unclear (Pettilä et al., 2011).

In body temperature measurements, we observed elevated values, confirming the presence of inflammatory responses in the body. In the septic group, both heart rate and aortic blood flow values were markedly elevated after 24 hours. These measurements confirm the clinical symptoms of sepsis (Font et al., 2020). The analysis of acid-base balance and blood gas parameters is essential both at the experimental and clinical levels (Ganesh et al., 2016). In our study, the measured increase in partial carbon dioxide levels and decrease in partial oxygen levels indicate impaired tissue oxygenation. Lactate and creatinine concentrations showed a significant rise compared to baseline values. Jingyi Wang and colleagues examined the method of blood gas analysis and observed how effective its application could be in clinical practice (Wang et al., 2023). Since the core of the condition lies in deteriorating microcirculatory processes, we should monitor parameters that reflect these changes.

The previously mentioned CytoCam-IDF device is one of the most modern instruments for the examination of the microcirculation, it enables fast, digital image processing, it has an automatic analysis software, which makes it possible to monitor PVD (perfused vessel density), PPV (proportion of perfused vessels), MFI (microvascular flow index), and the heterogeneity index (Guven et al., 2020). Even though it is still hard to use in the clinic and not very useful, there is a clear need for more reliable and easier-to-use methods. The UD Microcirculatory Video Score System (MVSS) was created at the Department of Operative Techniques and Surgical Research at the University of Debrecen. It is a semi-quantitative tool for assessing microcirculatory video recordings. The system looks at five important things: edema (0–2), red blood cell aggregation in both larger vessels and capillaries (0–3 for each), flow heterogeneity (0–3), and vascular morphology (0–2). Edema is rated from none to very high, and aggregation scores show how common and severe the problem is. Flow heterogeneity can be anything from normal, even perfusion to severe stasis with areas that aren’t perfused. Vascular structure can be anything from normal to very abnormal. These parts make up a whole. Together, these components form a composite score reflecting microcirculatory alterations.

Several scoring systems are used for the diagnosis and assessment of sepsis. The Sequential Organ Failure Assessment (SOFA) score is widely accepted, evaluating dysfunction across multiple organ systems (cardiovascular, respiratory, renal, hepatic, coagulation, and neurological) on a scale from 0 to 24, with higher scores indicating greater severity. While comprehensive and accurate, it requires extensive laboratory data. The Quick SOFA (qSOFA) score offers a simpler, bedside alternative based on three criteria (hypotension, tachypnea, and altered mental status), but it has limited sensitivity for early detection and less reliable prognostic value. The APACHE II–III scores provide strong mortality prediction based on multiple physiological parameters measured within 24 hours, though they are complex and not suitable for rapid bedside use (Churpek et al., 2017; Levy, 2006). In general, it can be said about the APACHE, SOFA, qSOFA scoring systems that examine several physiological parameters (e.g.: blood pressure, respiratory rate, body temperature, etc.), while the UD MVSS scoring system works in a completely different way from these.

Blood gas analysis reflects the balance between tissue oxygen supply and consumption and thereby serves as an indicator of the current state of microcirculation. Under normal conditions, lactate production and clearance are tightly regulated, resulting in typical levels around 2 mmol/L. When tissue oxygenation is inadequate, more lactate is produced through anaerobic metabolism. During sepsis, lactate levels rise; however, this can also result from other processes such as increased glucose utilization due to inflammation or enzyme activation leading to enhanced lactate production. Therefore, it is important to note that elevated lactate concentrations in sepsis may have multiple sources and are not necessarily caused solely by tissue hypoxia or hypoperfusion (Levy, 2006; Levy et al., 2008). Based on the study’s findings, blood gas analysis may serve as a reliable method for monitoring microcirculation, but these parameters should be interpreted in relation to the patient’s clinical condition and adjusted accordingly if necessary. The cecal ligation and puncture (CLP) we chose is accepted as the gold standard for sepsis studies. The CLP model can mimic human clinical symptoms, such as appendicitis and peritonitis, which can lead to fatal sepsis. During the design of the project and the performance of the experiment, we considered the recommendations and suggestions published in the literature (Lilley et al., 2015; Osuchowski et al., 2018). However, our study has some limitations. The experiment was short-term and included only two groups, focusing on acute changes in microcirculatory and hemorheological variables. Additionally, the study was conducted in rats with healthy vasculature; therefore, future research should explore different sepsis models and include treated groups to provide a more comprehensive understanding.

## 5 Conclusion

Sepsis is a complex and challenging clinical syndrome that is difficult to define due to its highly variable presentation. Over the years, researchers and clinicians have developed various scoring systems to support early recognition and enable timely initiation of treatment. These tools have improved clinical practice, but none of them is perfect, and sepsis continues to cause high mortality worldwide. Therefore, ongoing research focuses on better understanding the underlying mechanisms of the septic process and improving diagnostic accuracy. A key goal is to develop and implement more effective therapeutic strategies that can enhance patient outcomes and reduce the number of sepsis-related deaths.

## Data Availability

The original contributions presented in the study are included in the article/supplementary material. Further inquiries can be directed to the corresponding author.
